# A cross sectional study of maternal near miss and mortality at a rural tertiary centre in southern nigeria

**DOI:** 10.1186/s12884-017-1436-z

**Published:** 2017-07-28

**Authors:** Ikechukwu Innocent Mbachu, Chukwuemeka Ezeama, Kelechi Osuagwu, Osita Samuel Umeononihu, Chibuzor Obiannika, Nkeiru Ezeama

**Affiliations:** 1 Department of Obstetrics and Gynaecology, Nnamdi Azikiwe University Awka, Nnewi Campus, Nnewi, Anambra State Nigeria; 2 Department of Obstetrics and Gynaecology, Nnamdi Azikiwe University Awka, Nnewi Campus, Nnewi, Anambra State Nigeria; 3 Department of Obstetrics and Gynaecology, Madonna University Teaching Hospital, Elele, Rivers State Nigeria; 4 Department of Obstetrics and Gynaecology, Nnamdi Azikiwe University Teaching Hospital, Nnewi, Anambra State Nigeria

**Keywords:** Near miss indicators, Maternal death, Rural area, Private hospital

## Abstract

**Background:**

The study evaluated the pattern of severe maternal outcome, near miss indicators and associated patient and healthcare factors at a private referral hospital in rural Nigeria.

**Methods:**

This was a cross sectional study conducted from September 2014 to August 2015 in Madonna University Teaching Hospital Elele, Rivers State, Nigeria. Pregnant and postpartum women were recruited for the study using Nigeria near miss network proforma which was adopted from the WHO near miss proforma. We explored administrative, patient related and medical delays. Statistical analysis was done using SPSS version 20.

**Results:**

Of the 262 deliveries, 5 women died and 52 women had a near miss event. The maternal mortality rate was 1908/100,000. The maternal near miss mortality ratio was 11.4: 1 while the mortality index was 8.8%. Three out of the five deaths that occurred were in the age category of 20–24 years. Abortive outcome was the leading cause of maternal mortality contributing 2 of the 5 maternal mortality. The severe maternal outcome ratio was 218/1000 and maternal near miss incidence ratio was 198/1000. Hypertensive disorders of pregnancy contributed 16(28.1%) of the 57 cases with severe maternal outcome while Obstetrics hemorrhage and abortive outcome each contributed 14(24.6%). 6(10.5%) received treatment within 30 min of diagnosis while 19(33.3%) waited for greater than 240 min before they received intervention. There was a statistically significant association between time of intervention and final maternal outcome (*p*-value = 0.003). Administrative delay was noted in 20 cases, while patient related delay was noted in 44 cases.

**Conclusion:**

There is a high burden of near miss and unmet need for reproductive health services in rural areas of Nigeria. Different levels of delays abound and contribute to the disease burden. Periodic reviews will aid in elimination of the delays. There should be better communication between different levels of care and emphasis should be on early identification and referral of women for prompt management.

## Background

Although there has been some progress in Nigeria in reaching the maternal health Millennium Development Goals, there is still an urgent need to sustain and increase the quality, availability, and accessibility of maternal and child health commodities, given the failure to attain the standards set forth by the MDGs [1]. A recent report by World Health Organization showed that there has been slow reduction of maternal mortality in Nigeria from 1350/100,000 in 1990 to 1170/100,000 in 2000 and 814/100,000 in 2015 [1]. This is in contrast to United Nations Development fund for population report 0f 243/100,000 in 2014 [2]. However, the Nigeria demographic and health survey estimated the maternal mortality to be 576/100,000 [3]. These figures are mere estimates because of poor vital statistics and showed the burden of maternal mortality in Nigeria. This high rate of maternal mortality is closely associated with low utilization of reproductive health services and is highlighted in the Nigerian demographic and health survey (NDHS) 2013, where only 61% of pregnant women received antenatal care while only 38% of the deliveries are attended by skill birth attendants [3]. The survey also showed that women in rural areas contributed 70% of the home deliveries without supervision when compared to women in urban areas which contributed 30% of the home deliveries. This may be related to low educational level and socioeconomic status of women in rural area compared to women in urban areas which were observed in the survey.

The importance of maternal mortality as a vital indicator in assessment of health and other aspects of growth and development informed its inclusion as one of the targets in the 5th millennium development goal. Reducing worldwide ratios will definitely go a long way in improving maternal health. Multiple interventions like skilled birth attendance, adequate emergency obstetrics care coverage, family planning and antenatal care have all been shown to be effective in reducing mortality rates, and their implementation has helped better the lives of women particularly in sub-Saharan Africa [4]. However, absolute values for maternal mortality in hospitals are few which limit information surrounding the events. Mortality figures are by definition a negative endpoint irrespective of the interventions and this has led to the concept of maternal near miss.

The World Health Organization (WHO), define a maternal near miss as “a woman who nearly died but survived a complication that occurred during pregnancy, childbirth or within 42 days of termination of pregnancy” [5]. The Near Miss tool is a useful tool in assessment of Obstetrics care and can provide a lead to the cascade of events that result to maternal deaths. Because more numbers are studied and surviving women can tell their story when compared to maternal mortality, it helps to identify remote and immediate factors that are associated with maternal morbidities and mortalities. “It also permits the development of preventive and educational programs with improved allocation of resources in order to achieve a reduction of both maternal morbidity and mortality” [6]. Evaluations of maternal deaths and near miss cases provide opportunities to examine social, economic, and structural factors that increase the risk of maternal mortality and morbidities, and these findings can be used to plan interventions that are contextually appropriate. This will help in strengthening the health system for efficient management of cases.

The incidence ratio and other epidemiological parameters (which include mortality index, severe maternal outcome ratio and maternal near miss- mortality ratio) vary from region to region and country to country. Previously, different studies have used different criteria to determine the near miss rate. WHO has standardized the criteria to help in comparing, evaluation and implementations of programs targeted at reduction of maternal mortality and morbidities. David et al. noted a total near miss ratio of 20/ 1000 and maternal mortality ratio of 254/100, 000 and near miss fatality rate of 11.2/1000 in a study in Mozambique [7]. This is comparable to findings by Nelissen et al. in Tanzania (near miss incidence ratio of 23.6/1000, maternal mortality ratio of 350/100, 000, and near miss fatality of 12.9%) [8].

Oladapo et al. in a nationwide multicenter study in Nigeria recorded a total near miss ratio of 15.8/ 1000 and maternal mortality ratio of 1088/100, 000 [9]. The classical triad of delays (Delay 1: Deciding to seek care; Delay 2: Identifying and reaching the medical facility; Delay 3: Receiving adequate and appropriate treatment) propounded by Thaddeus and Maine [10] were noted and accompanied by several instances of inappropriate management consistent with findings by other studies [11, 12]. These delays are compounded by socio-cultural and economic factors. This is perhaps the first nationwide attempt to capture the actual near miss and maternal mortality in Nigeria. Previous data has relied on estimates which may not be very accurate thus leading to faulty planning and implementation.

However, there were two important omissions in their study. The centres were all located in the urban areas. The socio-demographic and medical characteristics of women in urban and rural areas may vary. Studies in Nigeria have shown that the proportion of skilled birth attendants are heavily weighted in favor of urban area [13]. Secondly, all the hospital were government institutions which account for only 23% of all deliveries in the country [3].

These hospitals are funded by the state and do not depend entirely on incomes generated by the hospitals. The characteristics of health indices in private versus public hospitals; rural versus urban areas may vary. Our study was aimed at evaluating the near miss and maternal mortality in a non-government tertiary health institution in rural Niger-Delta region of Nigeria using the Nigeria Near Miss protocol adapted from WHO Near Miss protocol [5, 14]. This will help to determine the pattern of severe maternal outcome, near miss indicators, patients and healthcare factors associated with these morbidities and mortalities in rural areas and private referral hospital in Nigeria.

## Methods

### Study design

This was a cross sectional study conducted from September 2014 to August 2015 in Madonna University Teaching Hospital Elele, Rivers State, Nigeria.

#### Study area

The Madonna University Teaching Hospital is a new private teaching hospital (founded in 2005 but became operational in 2009) and located in Elele, which is a rural area in Rivers State in South-South geopolitical zone of Nigeria. It serves as a referral centre for neighboring communities in Rivers State and Imo State. It offers comprehensive emergency care and other reproductive health services. The Hospital is owned by our savior Missionary of the Catholic Church. The Obstetrics and Gynecology department has 7 consultants, 4 medical officers, interns, midwives and other staff.

### Study population

The people of Elele are predominantly farmers and traders as is seen in most rural dwellers in southern part of Nigeria. The subjects in this study included women who died or suffered a maternal near miss from pregnancy, labor and puerperal complications based on WHO criteria.

### Method/study procedure

The subjects were recruited using an exhaustive sampling approach. Pregnant women admitted in the prenatal ward, labor ward and or in the accident and emergency ward, who survived a near miss or died were recruited for the study. Data was collected for a period of 1 year from September 1st 2014 to August 31st 2015. Two medical officers and five interns who had done or on rotation in Obstetrics and Gynaecology department were involved in data collection. The principal investigator supervised the medical interns and medical officers who were involved in data collection. On a daily basis a medical officer and an intern visited the Labor ward, Obstetrics ward, Gynecology ward, Accident and Emergency, Intensive care unit where the registers were used to recruit subjects for the study. The information obtained was continually updated until the discharge of the patient or death. The study proforma was adapted from the Nigeria Near Miss network which was previously published in a peer review journal with input from WHO Human Reproduction Programme Research Ethics [14]. Relevant information extracted include demographic and reproductive data, markers of organ dysfunction predisposing factors to the Near Miss or Maternal mortality. Primary and secondary causes of the severe maternal outcome event were collected. We also explored any administrative, patient related and medical delays in the course of the event and the definitive management targeted at the cause of the maternal near miss or death.

The study proforma did not contain the names of the subjects. This was done to maintain subjects’ privacy, confidentiality and anonymity. Informed consent from the subjects was not obtained because there was no personal contact with the patients and the data collectors. Ethical clearance was obtained from the ethics committee of Madonna University Teaching Hospital, Elele, Rivers state Nigeria before the commencement of the study.

Inclusion criteria. This is as shown in the Table 1.

**Table 1:** Inclusion Criteria

Criteria	Components
Severe maternal complications	Severe postpartum Hemorrhage
	Severe preeclampsia
	Eclampsia
	Sepsis or severe systemic infection
	Ruptured Uterus
	Obstructed labour
	Severe complications of abortion
Critical interventions or intensive care unit	Admission into intensive care unit
	Interventional radiology
	Laparotomy for severe obstetrics conditions
	Use of blood products (fresh frozen plasma etc.).
Life-threatening conditions (near-miss criteria)	Cardiovascular dysfunction{Shock
	cardiac arrest (absence of pulse/heart beat and loss of consciousness),
	Use of continuous vasoactive drugs
	cardiopulmonary resuscitation,
	severe hypoperfusion (lactate >5 mmol/l or >45 mg/dl), severe acidosis (pH <7.1)
Respiratory dysfunction	Acute cyanosis, gasping
	severe tachypnea (respiratory rate > 40 breaths per minute),
	severe bradypnea (respiratory rate < 6 breaths per minute)
	intubation and ventilation not related to anesthesia,
	severe hypoxemia (O2 saturation < 90% for ≥60 min or PAO2/FiO2 < 200)
Renal dysfunction	Oliguria non-responsive to fluids or diuretics, dialysis for acute renal failure,
	severe acute azotemia (creatinine ≥300 μmol/ml or ≥3.5 mg/dl)
Coagulation/hematological dysfunction	Failure to form clots, massive transfusion of blood or red cells (≥5 units),
	severe acute thrombocytopenia (<50,000 platelets/ml)
Hepatic dysfunction	–– Jaundice in the presence of pre-eclampsia,
	severe acute hyperbilirubinemia (bilirubin >100 μmol/l or >6.0 mg/dl)
Neurological dysfunction	Prolonged unconsciousness (lasting ≥12 h)/coma (including metabolic coma), stroke, uncontrollable fits/status epilepticus, total paralysis.
Uterine dysfunction	Uterine hemorrhage or infection leading to hysterectomy.

### Data analysis

The data generated was analsyed using SPSS version 20. There were cross tabulations and correlations to explore relationship. The *p*-value of <0.05 was used at a confidence interval of 95% (Appendix).

## Result

During the study period, there were 307 deliveries; 262 live births and 45 stillbirths. A total number of 57 severe maternal outcome was recorded; 52 women had a near miss event while 5 women died as a result of complications of pregnancy. This gives a severe maternal outcome ratio of 218/1000 live births and maternal near miss incident ratio of 198/1000. The maternal mortality rate was 1908/100,000 live births. The maternal near miss mortality ratio was 11.4: 1 while the mortality index was 8.8%.

Among the women with severe maternal outcome, 50/57 (91.2%) were currently married while 5/57(8.8%) were not married. Majority 55/57(96.5%) were Christians while 2/57(3.5%) were Muslims. Three out of the five (60%) of the maternal death was in the age category of 20–24 years. Table 2 and 3 shows the other socio-demographic characteristics of the patients and selected reproductive characteristics.

**Table 2:** Socio-demographic characteristics of the subjects

Variable	Frequency *N* = 57	Percentage	Near miss	Mortality
Age range				
< 20	2	3.5	2	0
20–24	11	19.3	8	3
25–29	19	33.3	18	1
30–34	18	31.6	17	1
35–39	7	12.3	7	0
≥ 40	0	0	0	0
Occupation				
Unemployed	21	36.8	19	2
Unskilled	17	29.8	16	1
Semi skilled	14	24.6	12	2
Professional	5	8.8	5	0
Educational level				
No formal education	2	3.5	1	1
Primary	16	28.1	16	0
Secondary	30	52.6	27	3
Post secondary	9	15.8	8	1
Marital status				
Married	52	91.2	48	4
Not married	5	8.8	4	1
Religion				
Christianity	55	96.5	50	5
Islam	2	3.5	2	0

**Table 3:** Selected reproductive characteristics of the subjects

Parameter	Severe maternal outcome	Percentage	Near miss	Maternal death	Mortality index
Total number of pregnancies	*N* = 57				
1	14	24.6	12	2	14.3
2–4	26	45.6	25	1	3.8
≥5	17	29.8	15	2	11.8
Booking status at our hospital					
Booked	12	21.1	11	1	0.08
Unbooked	45	78.9	41	4	8.9
Referral status					
Booked at the hospital	12	21.1	11	1	0.08
Not referred	27	47.3	25	2	7.4
Referred before labor	5	8.8	5	0	0
Referred during labor	10	17.5	8	2	20
Referred postpartum	3	5.3	3	0	0
Trimmester at presentation					
1	9	15.8	8	1	12.5
2	11	19.3	10	1	9.1
3	34	59.6	31	3	8.8
Postpartum	3	5.3	3	0	0

Mortality Index = Number of Mortality/Near Miss + Mortality per 100

Hypertensive disorders of pregnancy contributed 16(28.1%) of the severe maternal outcome (SMO) while Obstetrics hemorrhage and abortive outcome each contributed 14(24.6%) of the SMO. Eclampsia and severe preeclampsia were the most frequent disease entity each contributing 8(14%) of severe SMO.

Abortive outcome (early pregnancy bleeding) was the leading cause of maternal mortality contributing 2(40%) of the maternal mortality from ectopic pregnancy and abortion related haemorrhage. It also has the highest mortality index of 14.3%. The distribution of the primary cause of the SMO is shown in Table 4. The 5(100%) of the maternal mortality were due to direct obstetric causes. Eclampsia, ruptured uterus and obstructed labour each contributed to one maternal death.

**Table 4:** The distribution of primary causes of severe maternal outcome

Primary cause	Severe maternal outcome *N* = 57	Percentage	Near miss	Maternal mortality	Mortality index (100%)
Obstetrics hemorrhage	14	24.6	13	1	7.1
Placenta praevia	1	1.8	1	0	0
Abruptio placentae	3	5.3	3	0	0
Ruptured uterus	7	12.3	6	1	14.3
Postpartum hemorrhage	3	5.2	3	0	0
Infection	1	1.8	1	0	0
Peuperal genital tract sepsis	1	1.8	1	0	0
Hypertensive disorders	16	28.0	15	1	6.3
Severe preeclampsia	8	14.0	8	0	0
Eclampsia	8	14.0	7	1	12.5
Prolonged obstructed labor	10	17.5	9	1	10
Abortive outcome	14	24.6	12	2	14.3
Abortion related haemrrhage	5	8.8	4	1	20
Abortion related infection	2	3.5	2	0	0
Ruptured ectopic pregnancy	7	12.3	6	1	14.3
Severe malaria/anaemia	2	3.5	2	0	0

There was variable lag between time of diagnosis and intervention in most of the cases. Only 6(10.5%) received treatment within 30 min after diagnosis while 19(33.3%) waited for greater than 240 min before they received intervention. 4(80%) of the mortality were noted in the greater than 240 min group. There is statistical significant association between time of intervention and final maternal outcome (*p*-value = 0.003). This is shown in Table 5.

**Table 5:** Time interval between diagnosis and intervention in minutes

Time range	Frequency	Percentage	Near miss	Mortality	*P*-value
< 30	6	10.5	6	0	0.003
31–60	16	28.1	16	0	
61–120	7	12.3	6	1	
121–180	5	8.8	5	0	
181–240	4	7.0	4	0	
>240	19	33.3	15	4	
TOTAL	57	100	52	5	

Delays in management were noted in 46(80.7%) of all the cases. Administrative delays were noted in 20 cases and non-availability of blood products 7(12.3) being the leading problems. Patients related delays were noted in 44 cases. Late presentation 22(38.6%), inability to pay 10(17.5%) and lack of transportation 9(15.8%) were the most frequent patient related problems. Different delays are shown in Table 6.

**Table 6:** Patient and health institution delays

Administrative delay	Frequency(*n* = 57)	Percentage
No power supply	3	5.3
No transpaort and or communication	9	15.8
Non availability of blood/blood products	7	12.3
Absence/lack of equipment	1	1.7
No administrative problem	37	64.9
Total	57	100
Patient oriented problem	Frequency(*n* = 57)	Percentage
Late presentation	22	38.6
Refusal of treatment	12	21.1
Inability to pay	10	17.5
No patient related problem	13	22.8
Total	57	100
Medical personnel problem	Frequency(*n* = 57)	Percentage
Delay in treatment	20	35.1
No assessment by senior doctor	6	10.5
Poor monitoring	1	1.8
None	30	52.6
Total	57	100

## Discussion

Our study is one of the few studies in Nigeria that evaluated Near miss morbidities. To the best of our knowledge, this is the first study on near miss in a private health institution and in a rural area in Nigeria. This has highlighted the burden of severe maternal outcome in the rural settings in Nigeria. It also showed the gaps and strength of a tertiary private hospital in rural Nigeria.

The near miss incident rate of 198/1000 and SMO 218/1000 recorded in this study is higher than the values 16/1000 and 27/1000 recorded in Nigeria near miss network [9] and near miss ratio of 28.6/1000 recorded by Tuncalp et al. in Accra Ghana [15]. It is also higher than 28.6/10000 reported by Neilsson et al. rural settings in Tanzania [7]. Even far lower values have been recorded in developed countries [16, 17]. The reason may be because only few hospitals in rural areas of Nigeria offer comprehensive obstetrics emergency care. Most secondary health institutions in rural Nigeria offer poor services to the populace because of the weak health sector and lack of political will. These health institutions are characterized by poorly motivated staff due to poor remuneration and delay in payment of salary. Other contributing factors to poor services include lack of equipment and infrastructure and high attrition of staff to health institutions in urban areas.

In addition, most deliveries in rural areas occur at home while some women are managed by traditional birth attendants who may have a high threshold for referral of high risk women only when there is severe complication and impeding maternal death as evidenced low number of uncomplicated pregnancies managed in the institution during the study period.

The maternal mortality rate of 1900/100,000 is far higher than the national estimates of 547/100,000^3^. One of the reasons for this observation may be connected to the high percentage of severe cases. Lower rates of maternal mortality have been recorded in studies in other developing countries [18, 19].

The mortality index of 8.8% recorded implies that for every 10 patients that have severe maternal outcome, one is likely going to die from the complications of pregnancy. It is lower than 41% observed by Nigeria near miss network study which was carried out in public hospitals [10]. This may be related to more bureaucratic bottle necks in public hospitals in Nigeria and early involvement of consultants in the management of the cases in private hospitals. Similar values have been reported in studies in other developing countries [20]. A study by Adisasmita et al. also noted less mortality index in private hospitals compared to public hospitals [21].

The pattern of primary causes of near miss in this study mirrors observations of several studies with hypertensive disorders and hemorrhage being the leading causes [7–9, 11, 12]. However with respect to mortality, we recorded highest mortality index in the abortive/early pregnancy bleeding. This is different from several other studies where Eclampsia had the highest case specific mortality index [7–9]. This may be explained by the routine use of MgSO4 in our center which reduced the mortality associated with Eclampsia. The only patient that died from Eclampsia presented late with multiple organ dysfunction. A study in Sagamu, south west Nigeria where Eclampsia was the leading cause of mortality noted that most patients with Eclampsia did not receive MgSo4 which is consistent with observations from other studies outside Nigeria. [12].

Contrary to other studies, there was no mortality as a result of primary postpartum hemorrhage in our study [9, 11]. This may be explained by aggressive management and prevention of cases. Active management of third stage is offered routinely to all women that deliver in the Centre. In addition, we give prophylactic doses of misoprostol to high risk patients.

The case specific mortality index in our study was highest with early pregnancy bleeding (14.3%). It also contributed 24.7% of the severe maternal outcome. This is worrisome and will need further studies to determine the cause of high incidence and mortality. This may be connected with the characteristics of the patients which include young age, delayed recognition and awareness of complications, unmet need for contraception, high dependent level and lack of access to health facilities. The various levels of delays noted in our study may have contributed to the mortalities.

Significant delays were observed at various levels in this study. This is consistent with the Nigeria near miss network study observation and (WHO STUDY) [9, 17]. This means there is need to shift from quantity to quality of care to reduce mortality. The findings of no maternal death among women who received treatment within 30 min of presentation and 80% of the mortality occurring among those with greater than 240 min of delay before treatment implies that reduction of the time interval from diagnosis to intervention may reduce the mortality. This means that type three delay is a very potential target for reduction of maternal mortality in rural areas of Nigeria.

We infer that for substantive reduction in maternal mortality, the hospitals must institute mechanisms to reduce the type 3 delay. This includes twenty four hour emergency services without emphasis on fee for service in the few hours after presentation, partnering with government and other stakeholders in developing community health insurance services, and 24 h blood bank services. It also includes periodic evaluation of staff attitude to work. There is a need for periodic reviews to identify the different challenges in the management of women with severe maternal outcome.

The low utilization of a tertiary institution for routine reproductive health services is also a source of worry. This may be related to high patronage of unskilled birth attendants and home deliveries as evidenced by the high percentage of unbooked patients and late referral of women with life threatening pregnancy complications. The issue of unemployment and low education should be addressed to empower these women to make informed choices. There is need for increased awareness, reorientation of populace in rural settings to increase the uptake of EMOC in rural areas.

Our study has some limitations. This a single hospital studies in a rural setting, so there should be some caution in interpretation of the study. We did not ascertain the reasons for late presentations which would have aided in understanding factors remote from our Centre that endangered the life of these women. There is also need to follow up on the study and carry necessary intervention to reduce the lapses in the management of these women.

## Conclusion

This study clearly showed the high burden of near miss in rural areas of Nigeria and unmet need for reproductive health services. Any long and short term health planning should bear in mind the challenges and peculiarities of rural settings. There are few government hospitals in rural areas and most of them offer poor services as a result of poor motivation of staff and lack of equipment hence private hospitals has a huge potential in offering reproductive health services in the rural settings. The issues of delays must be addressed to reduce pregnancy related deaths in rural areas.

Private hospitals depend on fees for sustenance and collaboration with government and other stakeholders will help in reduction of the burden of severe maternal outcome. Health care financing is critical to any plan to increase uptake of EMOC and community insurance program will help to capture those women not included in the national health insurance scheme.

It also showed that pattern of mortalities and characteristics of these women differ from urban areas. Many women do not have access to reproductive health services including antenatal care. These should be made accessible and affordable.

Different levels of delays abound and contribute to the disease burden. The hospitals offering comprehensive EMOC should reduce the delays using different mechanisms to reduce mortality and complications in the management of these women.

There should be better communication between different levels of care and emphasis should be on early identification and referral of women for prompt management.

Critical infrastructure should be put in place and there should be incentives for healthcare personnel working in rural areas. Socio-cultural factors should be explored to increase utilization of health facilities in rural areas.

## Appendix

### Definition of maternal near-miss indicators {according to WHO]

#### Maternal near-miss (MNM)

This refers to a woman who nearly died but survived a complication that occurred during pregnancy, childbirth or within 42 days of termination of pregnancy.

#### Maternal death (MD)

This is the death of a woman while pregnant or within 42 days of termination of pregnancy or its management, but not from accidental or incidental causes.

**Live birth (LB)** refers to the birth of an offspring which breathes or shows evidence of life.

**Severe maternal outcome refers to** a life-threatening condition (i.e. organ dysfunction), including all maternal deaths and maternal near-miss cases.

**Women with life-threatening conditions (WLTC) refers** to all women who either qualified as maternal near-miss cases or those who died (i.e. women presenting a severe maternal outcome). It is the sum of maternal near-miss and maternal deaths (WLTC = MNM + MD).

**Severe maternal outcome ratio (SMOR)** refers to the number of women with life-threatening conditions (MNM + MD) per 1000 live births (LB). This indicator gives an estimate of the amount of care and resources that would be needed in an area or facility [SMOR = (MNM + MD)/LB].

**MNM ratio (MNMR)** refers to the number of maternal near-miss cases per 1000 live births (MNMR = MNM/LB). Similarly to the SMOR, this indicator gives an estimation of the amount of care and resources that would be needed in an area or facility.

**Maternal near-miss mortality ratio** (MNM: 1 MD) refers to the ratio between maternal near miss cases and maternal deaths. Higher ratios indicate better care.

**Mortality index refers** to the number of maternal deaths divided by the number of women with life-threatening conditions expressed as a percentage [MI = MD/ (MNM + MD)]. The higher the index the more women with life-threatening conditions die (low quality of care), whereas the lower the index the fewer women with life-threatening conditions die (better quality of care).

## Abbreviations

EMOC: Emergency obstetrics care
NDHS: Nigeria Demographic Health Survey
SMO: Severe Maternal Outcome
WHO: World Health Organization

## References

[1] Trends in Maternal mortality: 1990 to 2015. Estimates developed by WHO, UNICEF, UNICEF, UNFPA and The World Bank. Assessed at http://www.who.int/reproductivehealth/publications/monitoring/9789241502221/en/.

[2] Millennial development Goals. End Point Report-Nigeria 2015. http://www.sparc-nigeria.com/RC/files/4.2.20.2_Nigeria_MDG_Report_2015_Full_Report.pdf. Accessed 17 June 2017.

[3] Nigeria Demographic and health Survey 2013. National Population Commission, federal Republic of Nigeria, Abuja, Nigeria June 2014.

[4] Darmstadt GL, Maechant T, Claeson M, Brown W, Morris S, Donnay F, Taylor M (2013). A strategy for reducing maternal and newborn deaths by 2015 and beyond. BMC Pregnancy and Childbirth.

[5] Evaluating the quality of care for severe pregnancy complications. The WHO near-miss approach for maternal health 2011. http://www.who.int/reproductivehealth/publications/monitoring/maternal-mortality-2015/en/. Accessed 30 Apr 2013.

[6] Ronsmans C, Fillipi V. Beyond the numbers: reviewing maternal deaths and complications to make pregnancy safer. Geneva, WHO; 2004. p. 103–24.

[7] David E, Machungo F, Zanconato G, Cavaliere E, Fiosse S, Sululu C, Chiluvane B, Bergstrom S (2014). Maternal near miss and maternal deaths in Mozambique: a cross-sectional, region-wide study of 635 consecutive cases assisted in health facilities of Maputo province. BMC Pregnancy and Childbirth.

[8] Nelissen EJT, Mduma E, Ersdal HL, Evjen-Olsen B, van Roosmalen JM, Stekelenburg J (2013). Maternal near miss and mortality in a rural referral hospital in northern Tanzania: cross-sectional study. BMC Pregnancy and Childbirth.

[9] Oladapo OT, Adetoro OO, Ekele BA, Chama C, Etuk SJ, Aboyeji AP, Onah HE, Abasiattai AM, Adamu AN, Adegbola O, Adeniran AS, Aimakhu CO, Akinsanya O, Aliyu LD, Ande AB, Ashimi A, Bwala M, Fabamwo A, Geidam AD, Ikechebelu JI, Imaralu JO, Kuti O, Nwachukwu D, Omo-Aghoja L, Tunau K, Tukur J, Tuncalp Ӧ, Vogel JP, Gulmezoglu AM, Nigeria Near-miss and Maternal Death Surveillance Network. When getting there is not enough: a nationwide cross-sectional study of 998 maternal deaths and 1451 near-misses in public tertiary hospitals in a low-income country. BJOG. 2015; doi:10.1111/1471-0528.13450. 10.1111/1471-0528.13450 PMC5016783 25974281

[10] Thaddeus S, Maine D (1994). Too far to walk. Maternal mortality in context. Soc Sci Med.

[11] Jabir M, Abdul-Salam I, Suheil DM, Al-Hilli W, Abdul-Hassan S, Al-Zuheiri A (2013). Maternal near miss and quality of maternal health care in Baghdad. Iraq BMC Pregnancy and Childbirth.

[12] Oladapo TO, Sule-Odu AO, Olatuji AO, Daniel O. “Near-miss” obstetric events and maternal deaths in Sagamu, Nigeria: a retrospective study. Reprod Health. 2005;2, 9 doi:10.1186/1742-4755-2-9. 10.1186/1742-4755-2-9 PMC1291401 16262901

[13] Agbogoroma C. Distribution of Obstetricians and Gynaecology in Nigeria. Presented at the 49^th^ scientific conference of Society for Obstetrics and Gynaecology of Nigeria. Held at Asaba, Delta State 2014.

[14] Oladapo OT, Adetoro OO, Fakeye O, Ekele BA, Fawole AO, Abasiattai A (2009). National data system on near miss and maternal death: shifting from maternal risk to public health impact in Nigeria. Reprod Health.

[15] Tuncalp O, Hindin MJ, Adu-Bonsaffoh K, Adanu RM (2013). Assessment of maternal near-miss and quality of care in a hospital-based study in Accra, Ghana. Int J Gynaecol Obstet.

[16] Karolinski A, Mercer R, Micone P, Ocampo C, Mazzoni A, Fontana O (2013). The epidemiology of life-threatening complications associated with reproductive process in public hospitals in Argentina. BJOG.

[17] Souza JP, G€ulmezoglu AM, Vogel J, Carroli G, Lumbiganon P, Qureshi Z (2013). Moving beyond essential interventions for reduction of maternal mortality (the WHO multicountry survey on maternal and newborn health): a cross-sectional study. Lancet.

[18] Halder A, Jose R, Vijayselvi R (2014). Maternal mortality and derivations from the WHO near-miss tool: an institutional experience over a decade in southern India. J Turk Ger Gynecol Assoc.

[19] Morse ML, Fonseca SC, Gottgtroxy CL, Waldmann CS, Gueller E (2011). Severe maternal morbidity and near misses in a regional reference hospital Rio Janeiro. Rev Bras Epidemiol.

[20] Ali AA, Khojali A, Okud A, Adam GK, Adam I (2011). Maternal near-miss in a rural hospital in Sudan. BMC Pregnancy and Childbirth.

[21] Adisasmita A, Deviany PE, Nandiaty F, Stanton C, Ronsmans C (2008). Obstetric near miss and deaths in public and private hospitals in Indonesia. BMC Pregnancy and Childbirth.

